# Introduction of Chalcogenide Glasses to Additive Manufacturing: Nanoparticle Ink Formulation, Inkjet Printing, and Phase Change Devices Fabrication

**DOI:** 10.1038/s41598-021-93515-y

**Published:** 2021-07-12

**Authors:** A. Ahmed Simon, B. Badamchi, H. Subbaraman, Y. Sakaguchi, L. Jones, H. Kunold, I. J. van Rooyen, M. Mitkova

**Affiliations:** 1 Department of Electrical and Computer Engineering, Boise State University, Boise, ID 83725-2075 USA; 2 Comprehensive Research Organization for Science and Society (CROSS), 162-1, Shirakata, Tokai, Ibaraki 319-1106 Japan; 3 Idaho National Laboratory, Idaho Falls, ID 83415 USA

**Keywords:** Design, synthesis and processing, Electronic devices, Nanoparticles

## Abstract

Chalcogenide glasses are one of the most versatile materials that have been widely researched because of their flexible optical, chemical, electronic, and phase change properties. Their application is usually in the form of thin films, which work as active layers in sensors and memory devices. In this work, we investigate the formulation of nanoparticle ink of Ge–Se chalcogenide glasses and its potential applications. The process steps reported in this work describe nanoparticle ink formulation from chalcogenide glasses, its application via inkjet printing and dip-coating methods and sintering to manufacture phase change devices. We report data regarding nanoparticle production by ball milling and ultrasonication along with the essential characteristics of the formed inks, like contact angle and viscosity. The printed chalcogenide glass films were characterized by Raman spectroscopy, X-ray diffraction, energy dispersive spectroscopy and atomic force microscopy. The printed films exhibited similar compositional, structural, electronic and optical properties as the thermally evaporated thin films. The crystallization processes of the printed films are discussed compared to those obtained by vacuum thermal deposition. We demonstrate the formation of printed thin films using nanoparticle inks, low-temperature sintering and proof for the first time, their application in electronic and photonic temperature sensors utilizing their phase change property. This work adds chalcogenide glasses to the list of inkjet printable materials, thus offering an easy way to form arbitrary device structures for optical and electronic applications.

## Introduction

Additive manufacturing is one of the fastest developing industries since it offers compelling technology and an efficient device production process^1,2^, enabling roll-to-roll fabrication, which does not require cleanroom, photolithography and vacuum machinery. Moreover, the ability to print on any substrate type, including flexible substrates^3,4^, increases its application across electronics like the internet-of-things (IoT), wearables, sensing, and energy market. One other advantage of printing is that it can produce any arbitrary shape from a design file and the fabrication process is digitally controlled. So, printing is especially applicable to fabricate glass structures at a scale and complexity that were never possible before. However, most works utilizing such advantages are focused on printing fused silica glass^5–7^. There are other types of glasses, like chalcogenide glasses (ChGs), which are used as optical components and also for their electronic properties. ChGs are amorphous materials containing sulfur, selenium, or tellurium. These glasses are used as an active layer in sensors and memory devices. Among the published works on printing ChG, the most studied are filament molding type printing^8^ and dissolution-based inks^8–10^.

Inkjet printing is one of the most widely used technology in the field of printed electronics. It usually requires nanoparticle ink preparation that is compatible with a specific printer. However, solution based inks have also been successfully used in inkjet systems, notably for water soluble polymer patterns, to achieve superhydrophilic–superhydrophobic patterned surface^11^, and to fabricate high resolution metal patterns^12^. This paper describes a complete ChG printing process from glass synthesis to nanoparticle ink formulation and printing using a DMP-2850 Dimatix inkjet printer. ChGs have been a subject of immense interest for the past 5 decades after Ovshinsky patented ChG based memory devices^13^. This glass family is large since only the valence requirements of the participating atoms need to be satisfied to form a variety of glasses. Due to the lack of order, the atoms can be connected in many different configurations without stoichiometric restrictions. These glasses' unique properties stem from the fact that the chalcogen elements form lone pairs in their outer shell^14^ and use only the other two remaining *p* electrons for chemical interactions with the elements, building multiple selections of substances^15^. Such chemical property is beneficial to forming materials with high flexibility because the two-fold coordination of chalcogen atoms leads to the formation of a floppy structure^16,17^, providing ChGs with low Young's modulus^18^ useful for their application in flexible devices^19,20^.

So far, conventional deposition techniques like thermal evaporation, sputtering, or chemical vapor deposition have been used to fabricate ChG thin films. There are reports in the literature on solution-based ChG film deposition techniques, such as spin-coating^21–24^. However, this method does not allow the formation of devices with specific dimensions and shapes without expensive and complex photolithography. Furthermore, the solvents used in ChG solutions are usually amines^25–27^, which are highly toxic, corrosive and reactive. Usually, traces of these compounds remain in the deposited films and affect their properties. Considering digital printing of patterns using these solutions is not convenient since the amines react with the polymer housing inside printheads. This limits the application of so-produced inks due to the need to modify printers and/or the requirement of a controlled environment for the safe handling of the material. Nevertheless, some publications report devices printing with amine-based chalcogenide solutions^10^ using custom-made printers or syringe dispensation. New avenues must be explored to discover a better solution for applying additive technology for producing a wide range of electronic and optical devices based on ChG. The additive manufacturing would open enormous opportunities for devices production in space or other prospects like direct device printing over particular surfaces. In this respect, inks containing nanoparticles of ChGs are an unrivaled solution. While there are some reports in that field^28,29^, these inks have not been used in printers so far, and there are no reports regarding their capabilities for the production of electronic/photonic devices by printing.

This work is motivated to provide an alternative solution to traditional temperature sensing devices in Light Water or Sodium-cooled Fast reactors to measure temperature between 400 and 600 °C. ChG based materials transform from amorphous to crystalline state at a certain temperature. Due to the phase change, these materials alter their physical, electronic and optical properties and this effect can be utilized to measure temperature. Ge_x_Se_100−x_ (x = 30, 33, 40) ChGs, have crystallization temperature within the temperature range of importance (400–600 °C). Although, the concept of a phase change temperature sensor has around for some time^30^, the material was limited only to Ge_2_Sb_2_Te_5_, fabrication of such devices was done by traditional methods, and the application temperature was low. Moreover, Ge–Se materials have certain radiation hardness^31^ that proved them to be applicable in a high radiation environment.

In this work, we investigate nanoparticle ink formulation of three glasses, the Se-rich Ge_30_Se_70_, the stoichiometric Ge_33_Se_67_ and the Ge-rich composition Ge_40_Se_60_ of the Ge–Se system, demonstrate the formation of thin printed films produced from the ink and determine their use in the fabrication of electronic and photonic temperature sensors. We report data regarding the dependence of the size of the nanoparticles from the milling process and the essential characteristics of the formed inks, like contact angle and viscosity. The properties and crystallization processes of the printed films are compared to thermally evaporated thin films and discussed based on their compositional specifics. The application of printed thin films and dip-coated fibers is demonstrated for temperature sensors utilizing the phase change effect. The change in material properties due to phase transition is measured by collecting electronic or optical signals and interpreted as a function of the printed films' specifics.

## Results

### Chalcogenide glass ink formulation

Nanoparticle inks are prepared by crushing bulk glasses into nanoscale particles. Ge_x_Se_100−x_ (x = 30, 33, 40). Bulk glasses are synthesized by the process described in our previous work^32^. Bulk glassy material is crushed into smaller pieces using wet milling and ultrasonication, respectively, to make nanoparticles. The ink is essentially, ChG nanoparticles suspended in a liquid, where the liquid medium is cyclohexanone. In addition, a surfactant, here ethylcellulose, is added into the mixture to prevent particle agglomeration. Ethylcellulose readily dissolves in cyclohexanone, and the boiling point of cyclohexanone is high enough (155.6 °C) to avoid printers’ nozzles’ drying during printing. So, a mixture of cyclohexanone, ethylcellulose and ChG powder is milled, ultrasonicated and centrifuged, respectively. The milled mixture comes out as a highly viscous liquid. Cyclohexanone is added before ultrasonication to make it less viscous. Once the process is finished, the concentration of the cyclohexanone and ethylcellulose is adjusted to make the mixture viscosity compatible with Fujifilm Dimatix Material Ink-Jet Printer (DMP-2850) and then the mixture becomes an ink. Moreover, the ink drop's contact angle on the oxidized-silicon substrate is measured to estimate the resolution.

Particle size is an essential characteristic, closely controlled during ink preparation. A dynamic light scattering (DLS) system was used to measure the particle size distribution. The inks’ quality were characterized by their viscosity, i.e., their potential to be ejected by the printer, the ink surface tension and the surface energy of the substrate the inks are deposited on.

### Nanoparticle formulation and characterization

The process starts with ball milling. It has the provision to control the temperature during milling. Using that, the temperature was kept below 50 °C. Temperature control is imperative during milling as it prevents undesired crystallization of the nanoparticles. Figure 3 demonstrates that about 80 h of milling at 1100 rpm provides the optimum particle size (~ 200 nm) out of the ball mill. Further milling increases the particle size (~ 250 nm) as the milling and coalescence co-occur during extended milling. During milling, ethylcellulose reduces particle agglomeration rate and wastage of material by preventing particle adhesion to the milling jar and milling balls.

Increasing the ball milling's rotational speed might pin down the balls, reducing the effect of milling over particles. Also, high speed yields a high temperature that might crystallize the nanoparticles^33^. Although the ball mill temperature control is a helpful option, it controls the milling jar temperature rather than the local nanoparticles temperature. Moreover, milling balls might break, and the residues might contaminate the sample^34^. The trial-and-error studies demonstrated that for Ge_x_Se_100−x,_ an 1100 rpm rotation speed produces the best result. The final particle size was about 200 nm. After ball milling, the mixture comes out with paste-like viscosity. Another 50 ml of cyclohexanone is added to the paste to prepare a less viscous solution.

Ultrasonication is performed for 2.5 h to reduce the particle size further and disperse chalcogenide glass in the mixture. The ultrasonicator's electronic generator transforms AC line power to a 20 kHz signal that drives a piezoelectric transducer. The vibration is then amplified, which transmits down the length of a probe. The tip submerged into the sample expands and contracts. Due to the rapid vibration of the tip, it causes cavitation, the formation and collapse of minuscule bubbles in the liquid. The breakdown of thousands of bubbles releases tremendous energy in the liquid. Objects and surfaces within the liquid are thus "processed". The probe tip diameter dictates the amount of sample that can be effectively processed. Smaller tip diameters (Microtip probes) deliver high-intensity sonication, but the energy is focused within a small, concentrated area. As a result of ultrasonication, the DLS measurements demonstrated a considerable reduction in diameter to about 145 ± 20 nm.

**Figure 1:**
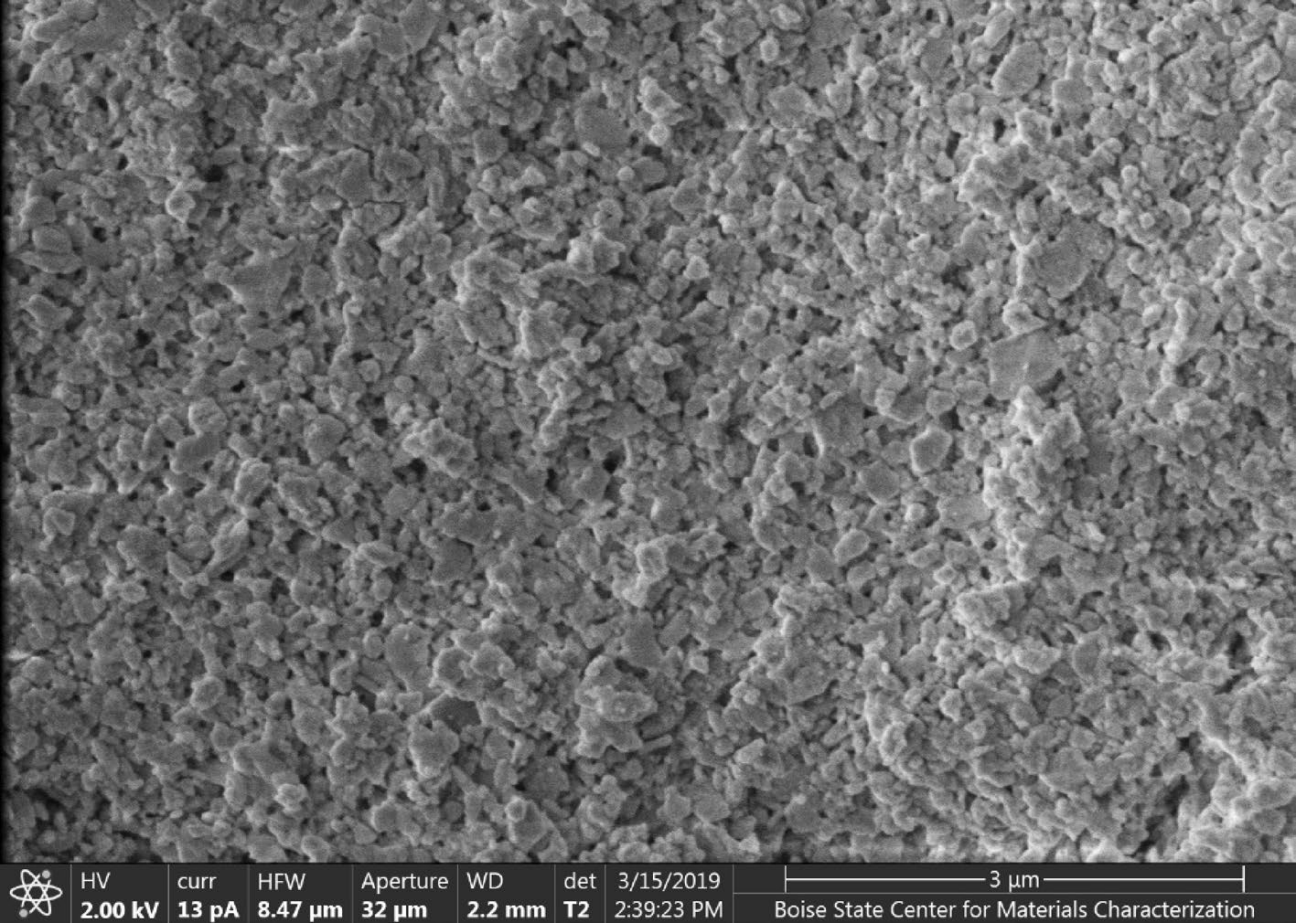
SEM micrograph of nanoparticles. Taken from an as-printed thin film.

Particle size uniformity in the mixture is achieved through centrifugation at 4500 rpm for 1.5 h, leading to the segregation of particles with a diameter of about 100 nm. Figure 1 is an SEM micrograph, showing the distribution of the nanoparticles on an as-printed thin film.

**Figure 2:**
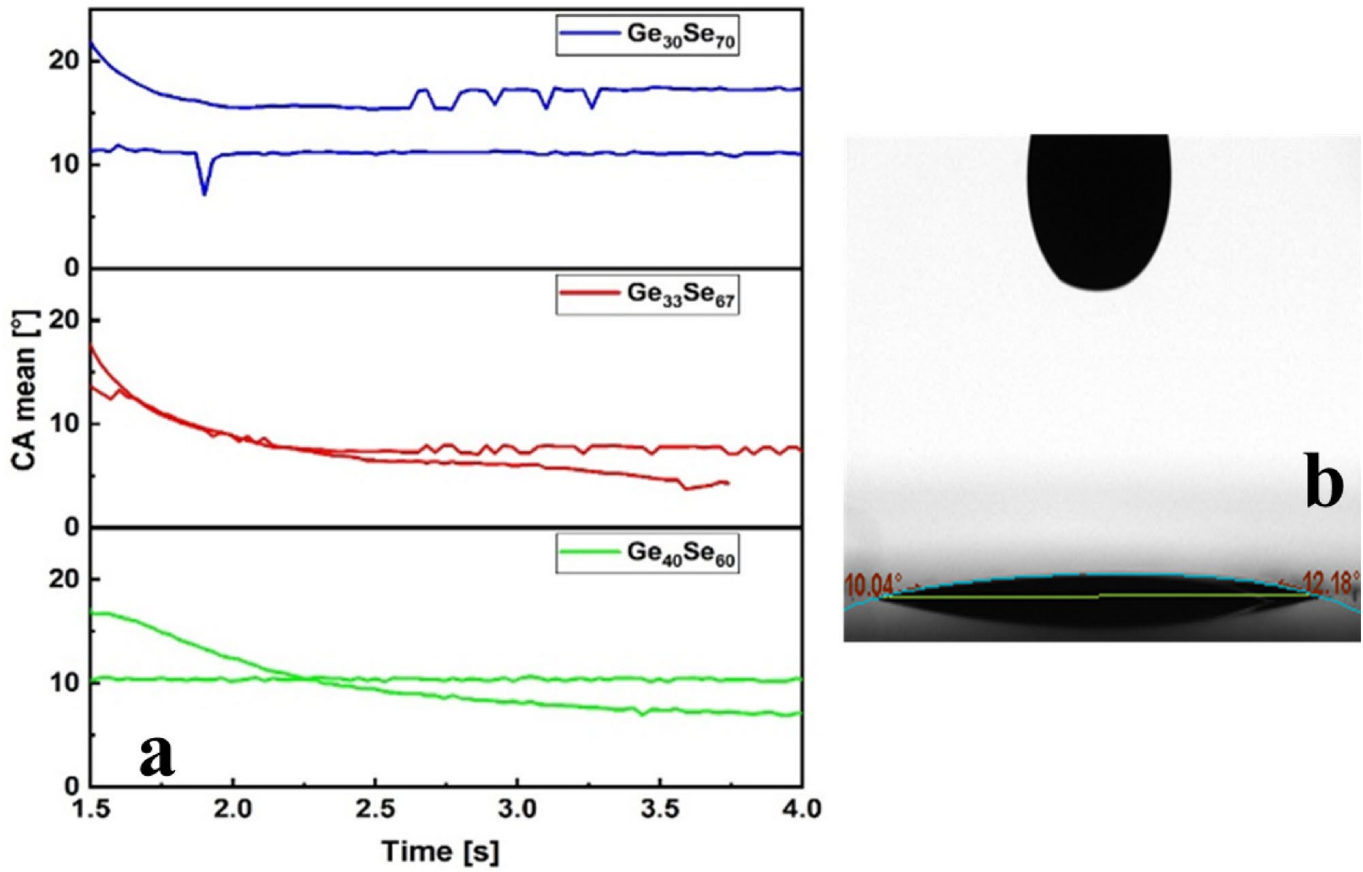
Contact angle measurement (**a**) mean contact angle, (**b**) actual measurement of Ge_30_Se_70_ ink on a thermal silicon dioxide layer.

Once the particle size is around 100 nm, the viscosity of the mixture is measured. The viscosity of the mixture needs to be within 8–12 cP to be compatible with the DMP-2850 printer. For final adjustment of the mixture viscosity, cyclohexanone and ethylcellulose were added to the milled mixture to prepare a compatible ink of viscosity 10–12 cP.

After viscosity, the ink's contact angle on the substrate (oxidized Si) is measured, which is an indicator of the adhesion and the resolution which can be achieved with the ink. All three ChG compositions showed contact angle 10–15°, suitable for good surface wettability. Figure 2 shows contact angle measurement of inks on oxidized silicon wafer. The inks' final concentration was 0.15–0.3 g/ml ChG and 0.03–0.05 g/ml ethylcellulose in cyclohexanone.

**Figure 3:**
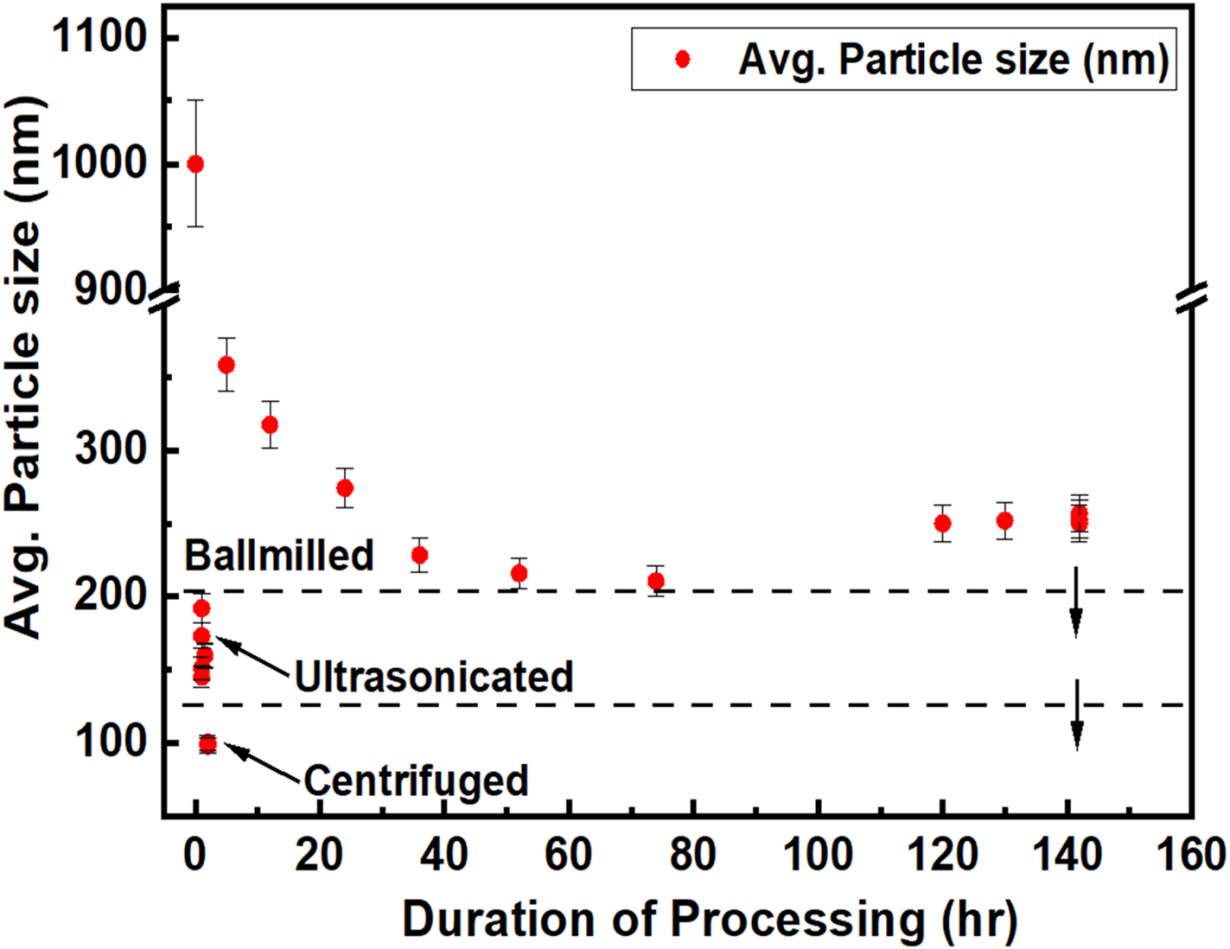
Particle size reduction of ChGs by combining ball-milling, ultrasonication, and centrifugation, respectively.

In the DMP-2850 printer, the printing was done using 3–5 nozzles. Nozzle voltage was between 20 and 30 V. The drop separation was set at 20 µm. Under such conditions, 100 µm resolution was achieved. For characterization, 10 layers of 5 cm × 5 cm thin films were printed.

### Printing and sintering

#### Printing

Although the surfactant concentration was optimized to have stable ink, over time the ink properties change, mostly nanoparticle agglomeration takes place. During printing, the ink is stationary and that might cause sedimentation. To have uniform performance of the ink over at least 2 days, there are some steps that are followed before printing to prepare cartridge and printhead.

1. Before printing, the inks were ultrasonicated (probe) in a test tube for 5 min, to have better dispersion and breakup some of the agglomeration.
2. Once the ink is inside the cartridge, the cartridge was put in an ultrasonic bath to remove any trapped gas and bubbles. This further disperses the nanoparticle.
3. Droplet velocity and shape were monitored through the “drop watcher” option of the Dimatix. Based on the duration of the droplets come out uniformly, a cleaning cycle was set. For example, if the 4 nozzles work accurately for 4 min, during printing a cleaning cycle was set to run every 3.5 min. During cleaning cycle, any blockage of the nozzles is removed.
4. Ultrasonic cleaning of the printhead to dislodge any particles clogging.

Moreover, the surfactant concentration was optimized to have stable ink. With the practice mentioned above, the inks were stable for 7 days.

#### Sintering

After printing, the printed films are wet and the nanoparticles are mixed with the surfactant. The printed films were dried for 2 days in a vacuum chamber at room temperature for the initial slow evaporation of cyclohexanone to avoid cracks formation. Once dry, the thin films were annealed at 350 °C (the decomposition temperature of ethylcellulose) for 2–3 h under nitrogen. Annealing sinters the particles and hardens the features forming solid printed films. After sintering, the printed films were characterized.

### Printed films characterization

#### Surface roughness

**Figure 4:**
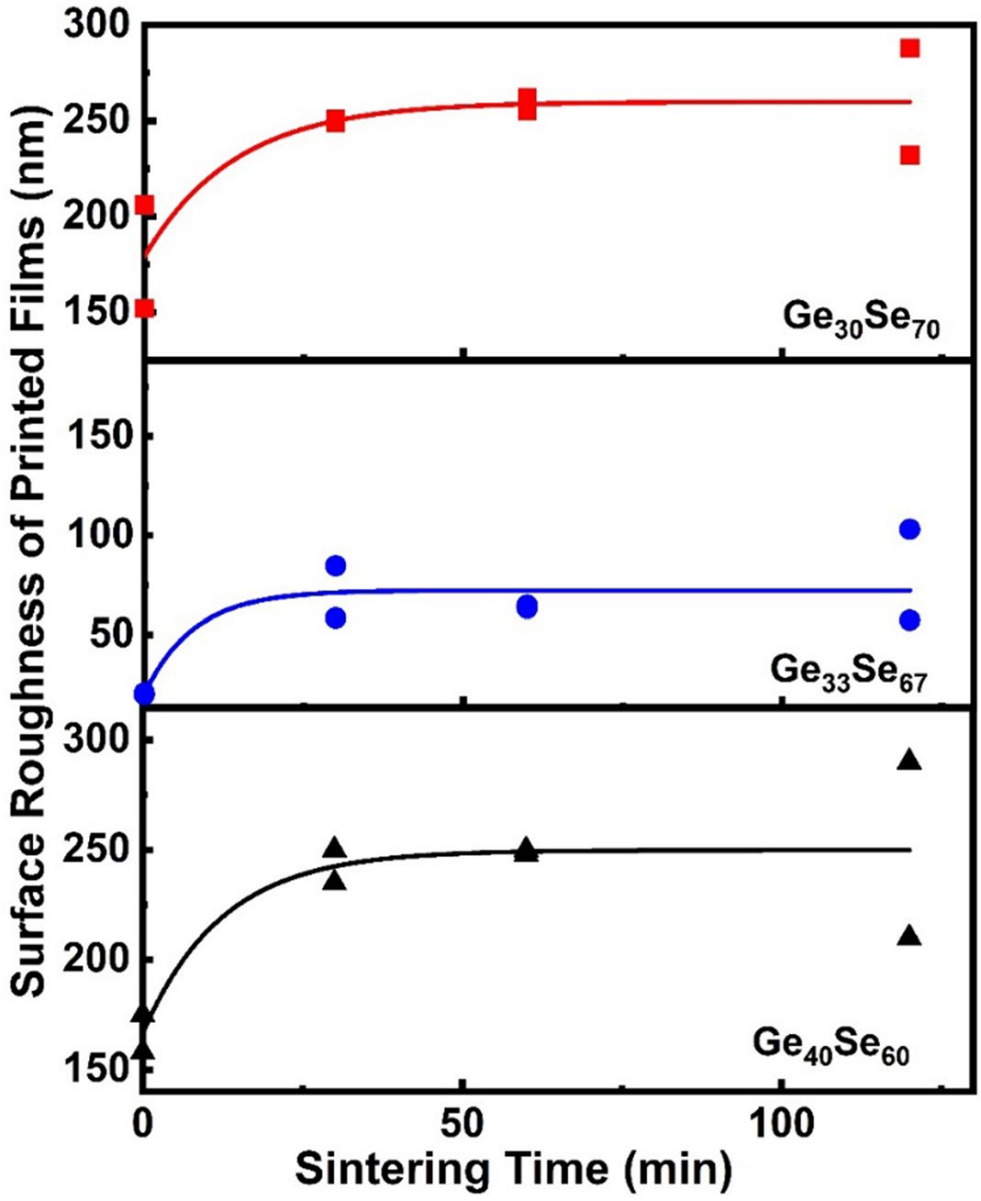
Average arithmetic roughness of the printed films as a function of sintering time.

The sintering duration and temperature causes macrostructural changes in the films and affects the films' surface roughness. The development of surface roughness as a function of the sintering time is presented in Fig. 4. The surface roughness increases during the initial phase of sintering (30 min of heating). However, further annealing does not influence the roughness much, and the curves display saturation, indicating that the sintering conditions are well satisfied. The curves in Fig. 4 are produced by non-linear fit. R^2^ values are for x = 33 − 0.7551, x = 30 − 0.74774 and x = 40 − 0.7396.

#### Compositional analysis

**Figure 5:**
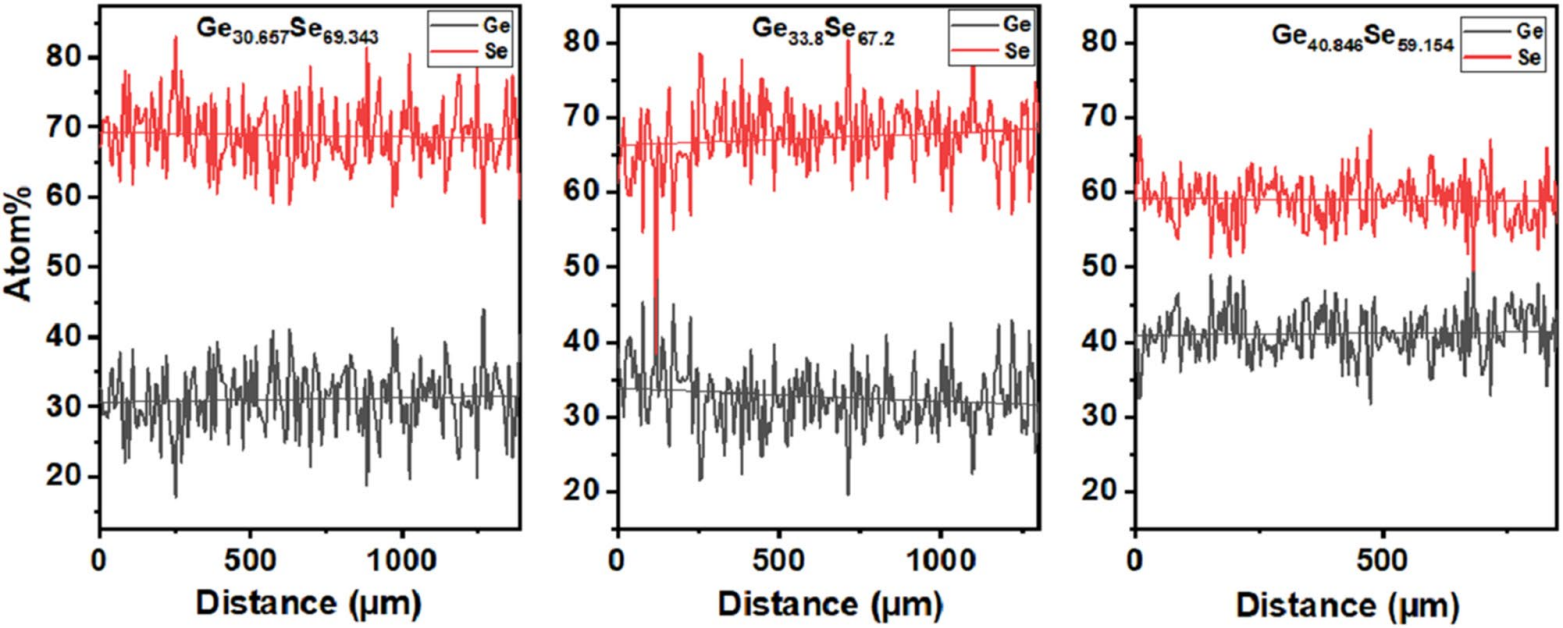
Line scan EDS of Ge_30_Se_70_, Ge_33_Se_67_ and Ge_40_Se_60_ printed films.

Since the ink contains two components (Ge and Se) with different hardness, there is a concern about the films' composition and structural stability. Characterization of printed thin films by Energy Dispersion Spectroscopy (EDS) shows that, on average, the composition of 5–6 μm thick films (Supplementary Information 1) obtained by ten layers of printing is close to the source bulk glass compositions. Figure 5 presents EDS line scans data of the printed films. Although point EDS shows a 3–5% difference in composition compared to the bulk glass, the average of 500–1000 µm line scan has less than 1% difference in composition from the bulk material. The film's composition checked by line scans is close to the bulk material composition, which assures a good printed film composition control.

#### X-ray diffraction spectroscopy

Further film characterization has been done by X-ray diffraction spectroscopy (XRD). Although, films with 30 at.% and 40 at.% Ge were amorphous in nature before and after sintering, the stoichiometric films with 33 at.% Ge were crystalline before sintering. After heating to their crystallization temperatures, the crystalline GeSe_2_ phases appearing in printed films with x = 30 and 33 are difficult to distinguish. The strongest peaks are from either *orthorhombic*-GeSe_2_ or *monoclinic*-GeSe_2_. According to the JCPDS (Joint Committee on Powder Diffraction Standards) card 32-0410, *orthorhombic*-GeSe_2_ shows the strongest peak near 14.93°, whereas the JCPDS card 30-0595 states that *monoclinic*-GeSe_2_ shows the strongest peak at 14.99°. So, the *orthorhombic* peak is seen at a slightly lower angle than *the monoclinic.* In the printed films, in samples with x = 30, the strongest peak was found at 14.96° and in those with x = 33, at 15°. From the experimental results, it can be inferred that unlike thermally evaporated films^32^, the x = 30 thin films crystallize forming *orthorhombic*-GeSe_2_ and such with x = 33 forms *monoclinic*-GeSe_2_. However, a 0.04° difference in the peak position could also be attributed to experimental error. Later the analysis of Raman data further enlightens the crystalline structure.

**Figure 6:**
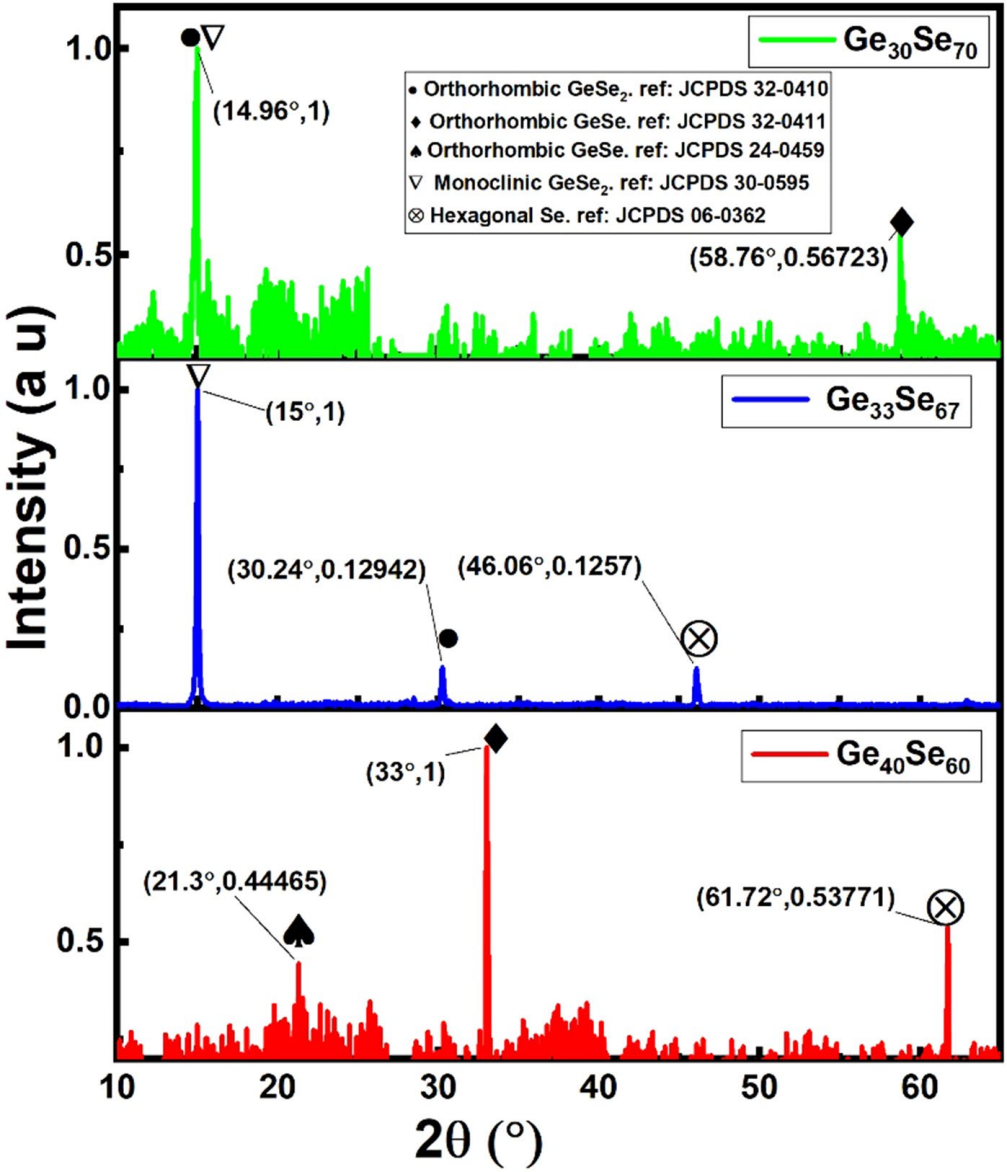
XRD of crystalline phases appearing after annealing of the films to the glass crystallization temperature.

In addition, the samples with x = 30 show the presence of GeSe crystals in the printed films, which could be due to phase separation occurrence since XRD also shows a well-documented presence of hexagonal Se crystals, as seen in Fig. 6. On the other hand, the samples with x = 40 display *orthorhombic*-GeSe, which agrees with the previous study^32^. As in the case for samples with x = 30, for both printed and thermally evaporated films, for x = 33, 40 compositions, *hexagonal*-Se is present. XRD shows no evidence of GeO_2_ or SeO_2_. The latter is very volatile and if formed, it should evaporate during sintering which was carried out at a temperature higher than its melting temperature (315 °C)^35^.

#### Raman spectroscopy

**Figure 7:**
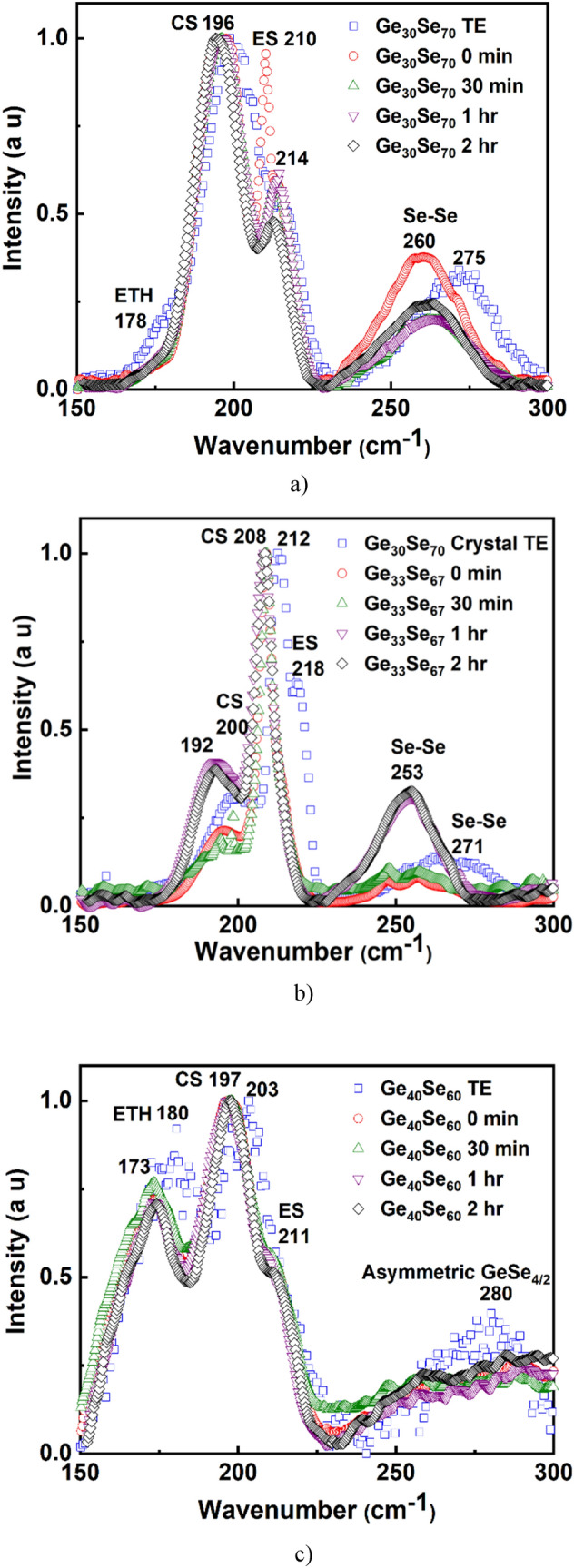
Raman spectroscopy of printed and evaporated films (**a**) Ge_30_Se_70_, (**b**) Ge_33_Se_67_, (**c**) Ge_40_Se_60_.

The molecular structure of the films was studied by Raman spectroscopy—Fig. 7a–c. The spectra of the printed films are compared to those of thermally evaporated (TE) films since it is known that the evaporated films closely resemble the Raman spectra of the bulk materials with the particular composition^36^. In essence, all electronic and photonic devices reported so far are based on thin films. Although all specific selenium and germanium containing tetrahedral structural groups (corner-sharing *CS*, edge-sharing *ES*, ethane-like *ETH*, and *Se–Se* chain) are present^37,38^, the Raman spectroscopy shows the difference in the structure of the printed, compared to thermally evaporated films. Since the samples with x = 33 crystallized during milling-ultrasonication, they will be discussed separately in the discussion.

Printed films with x = 30 show an increase in the *ES* structure, compared to the TE films—Fig. 7a. Once they are sintered at 350 °C for 30 min, printed films display similar structures as amorphous TE (a-TE) films. Further heating does not seem to affect their structural organization.

Compared to films with x = 30, those with x = 40 have shown better stability in terms of structural units—Fig. 7c. As expected, printed films exhibit a lower number of *ETH* structures than TE films, and there is a considerable red-shift of the *CS* and *ETH* peaks. A critical aspect of the films with x = 40 is that, even the "as printed" films before further annealing have similar structures as a-TE film.

## Discussion

Surfactant-assisted ball milling has been studied in-depth as it prevents particle agglomeration during milling^39–41^. Surfactants lower the surface energy of the fine particles during milling by forming a thin organic layer. The long organic tail of the surfactant prevents particles from coming in contact with each other. In this manner, it prevents the particles from agglomeration and cold welding that would substantially increase particle size during high-energy ball milling^42^. Concentration of the surfactant is an essential factor. For the ChG ink, 5% surfactant produces the best result. Although adding surfactant helps the milling, more than 5% concentration makes the thin films polymer-like and it reduces the adhesion to the substrate. Initially, the increasing of the milling time reduces the particle size, as presented in Fig. 3, any milling beyond 80 h tends to increase it. These nanoscale particles have a high surface/volume ratio. The bigger surface area creates a high number of dangling bonds. The particles become highly chemically active. Such reactivity contributes to their interaction and formation of agglomerates. We suggest two additional reasons for the formation of larger particles after a longer milling process. First, the high collision frequency between particles and grinding media reduces the surfactant's molecular chain to an average shorter length, thus decreasing the steric hindrance. Second, the high collision activity of the particles induces cold welding during the high-energy milling. These two steps produce a large mean agglomerate size in long-time milling^43^.

One of the significant challenges during the ink preparation-printing-sintering is the likelihood of oxides formation. However, their presence was not detected by the Raman and XRD studies, and they did not affect the performance of the device based on printed films.

One can expect that the milling process as described could lead to particle crystallization. The temperature control of the ball mill can only sense the temperature of the jar. However, during milling, localized heating (e.g., the heat generated due to the collisions between ChG–milling balls, milling balls–milling balls and milling balls–milling jar) occurs. This local temperature can be better maintained by reducing milling speed and by intermittent milling. Since reducing milling speed will produce larger particles, intermittent milling was done (30 min ON–30 min OFF). There are data^44,45^ that milling might further amorphize the material due to the mechanical stress over the crystalline structure. Besides, since the heat generation occurs fast and is localized, it might dissipate at an equal rate. Such a phenomenon emulates the "melt-quenching technique" that is used for glass formation. So, milling at the described conditions is expected to result in crystal-free glass nanoparticles as revealed by the Raman and XRD studies for the Ge_30_Se_70_ and Ge_40_Se_60_ samples. However, crystalline structure has been found in the as-printed films of stoichiometric composition Ge_33_Se_67_. Interestingly, this material, as we found out in our earlier study^32^, undergoes homogeneous crystallization, which requires more energy than the heterogeneous process characteristic for the non-stoichiometric compositions. Apparently, the milling introduces enough energy for the crystallization to occur. Since, in this case, the number of the wrong Ge–Ge and Se–Se bonds is minimal, and the requirement for the lower enthalpy for this composition is critical, crystallization dominates.

Moreover, printed crystallized x = 33 thin films show similar Raman spectra of thermally evaporated crystallized x = 30 thin films. Ball-milling introduces phase separation in the nanoparticle which in turn changes the crystallization kinetics of x = 33 from homogenous to heterogenous. In addition, Fig. 5 shows x = 33 thin films are slightly Se-rich. We suggest both Se-rich nature and phase separation dominates the crystallization of printed x = 33. As a result, the crystal phases are different than thermally evaporated thin films of x = 33.

As pointed out in the XRD results—Fig. 6, it is difficult to decide which polymorph form of GeSe_2_ has crystallized. The Raman studies of the material with x = 33 *CS* at 208–212 cm^−1^ indicates the formation of *orthorhombic*-GeSe_2_^38^ and *CS* at 192–200 cm^−1^ suggests the formation of *monoclinic*-GeSe_2_. So, from Raman spectroscopy, we can infer that the peak at 15° in samples with x = 33 Fig. 6 is from *monoclinic*-GeSe_2_.

From an application point of view, printed features with a similar material structure as the bulk glass or thermally evaporated films are essential. The scope of this paper is confined to the study and comparison of printed and TE films. Although all specific germanium containing tetrahedral structural groups (CS, ES, and ETH) are present^32^, the Raman spectroscopy shows two significant differences between printed and TE films. The first is related to the reduction or absence of *ETH* structural units around 178 cm^−1^^37^ in printed films before temperature annealing. ChGs are chemically disordered^46^ materials, which means there is a presence of wrong bonds like Ge–Ge and Se–Se. Ideally, in samples with x = 30, Se–Se bonds are expected since they are Se-rich. But the presence of Ge–Ge bonds is widely accepted for this composition as well due to the disordered character. However, Ge–Ge bonds are weakest compared to Ge–Se and Se–Se bonds. So, we suggest Ge–Ge bonds break during the milling. Second, there is a red-shift of the *CS* and *Se–Se* peaks in the printed films. The effect is much prominent in the *Se–Se* peak (almost 15 cm^−1^). *CS* peaks seem to undergo red-shift with sintering duration. Although ultrasonication uses sound waves of wavelength in cm scale, such stress causes change at Å scale. Adding mechanical stress and high pressure during milling introduces additional distortion of the bonding^47^. Compared to the TE films, in all printed films, all the peaks demonstrate a red-shift, which should be related to the material being Ge deficient^37^ except *ES* in samples with x = 30. As Fig. 5 displays, samples with x = 30 and 40 are a bit Ge-rich than corresponding bulk materials. However, we suggest that there could also be another mechanism dominating the peak position other than the composition. It has been shown that when a material undergoes tensile stress, the chemical bonds might get elongated relative to their unstressed state^47^. As the bond length increases and the force constant remains the same, it is expected that the vibrational frequency will decrease. During nanoparticle formation from bulk, printing and sintering, the material is subjected to various mechanical stresses. Stress in the material and composition effectively influences the Raman peak positions, causing a red-shift and is the reason for the difference in the crystalline phases occurring after annealing of printed films. This effect also demonstrates the multifaceted character of the mechanical interactions in ink formation during milling and printing unlike the one-directional pressure effect, which compresses interatomic bonds and causes a blue-shift to the vibrational modes due to anharmonic effects^48^.

The sintering time is also a factor influencing the films' structure. For example, the missing *ETH* like structure, especially in the Se-rich films, stabilizes with increasing the sintering time. A similar effect is characteristic of the edge-sharing structures and selenium chains. The Ge–Ge bond has the lowest bond strength in the studied system, and its presence is mainly affected by the milling process. For this reason, the Ge–Ge bonds have a relatively limited appearance on the Raman spectra of the printed chalcogen-rich films. Regarding the stoichiometric composition which initial crystalline structure is based mainly on monoclinic GeSe_2_ the long-term annealing relaxes the material through phase separation by which wrong bonds Ge–Ge and Se–Se dominate the structure. The Raman spectra shows improved and manifested structure closer to one of the TE films with the increase in sintering time due to decomposition of all additives used to form the ink and structural relaxation and thus bring the material to its equilibrium condition and stable structure. A similar result has been submitted by Slang et al.^49^ for films obtained through the dissolution of ChG. It should be noted here that the post-processing mechanism of spin-coated films, prepared using dissolved glass and nanoparticle ink printed films, is different, although they both involve annealing. Spin-coated films require low-temperature annealing to evaporate the solvent, which has a low boiling temperature. In nanoparticle films, the sintering must be carried out at a higher temperature to decompose surfactants and, in some cases, to melt the nanoparticles. These specifics of the sintering have been reported for dissolved As–S glasses^23^ and As–Se glasses^50,51^. However, the sintering process negatively affects the films' surface roughness, as shown in Fig. 4. We suggest that this is a result of particles' agglomeration and reduction of film thickness after evaporation of the solvents and decomposition of surfactant^52^.

## Application

Although the printed films' application is possible in different electronic or optical memory devices, examples will be discussed related to temperature sensors based on electrical and optical data collection, which have been first developed by our group.

### Electronic temperature sensor

**Figure 8:**
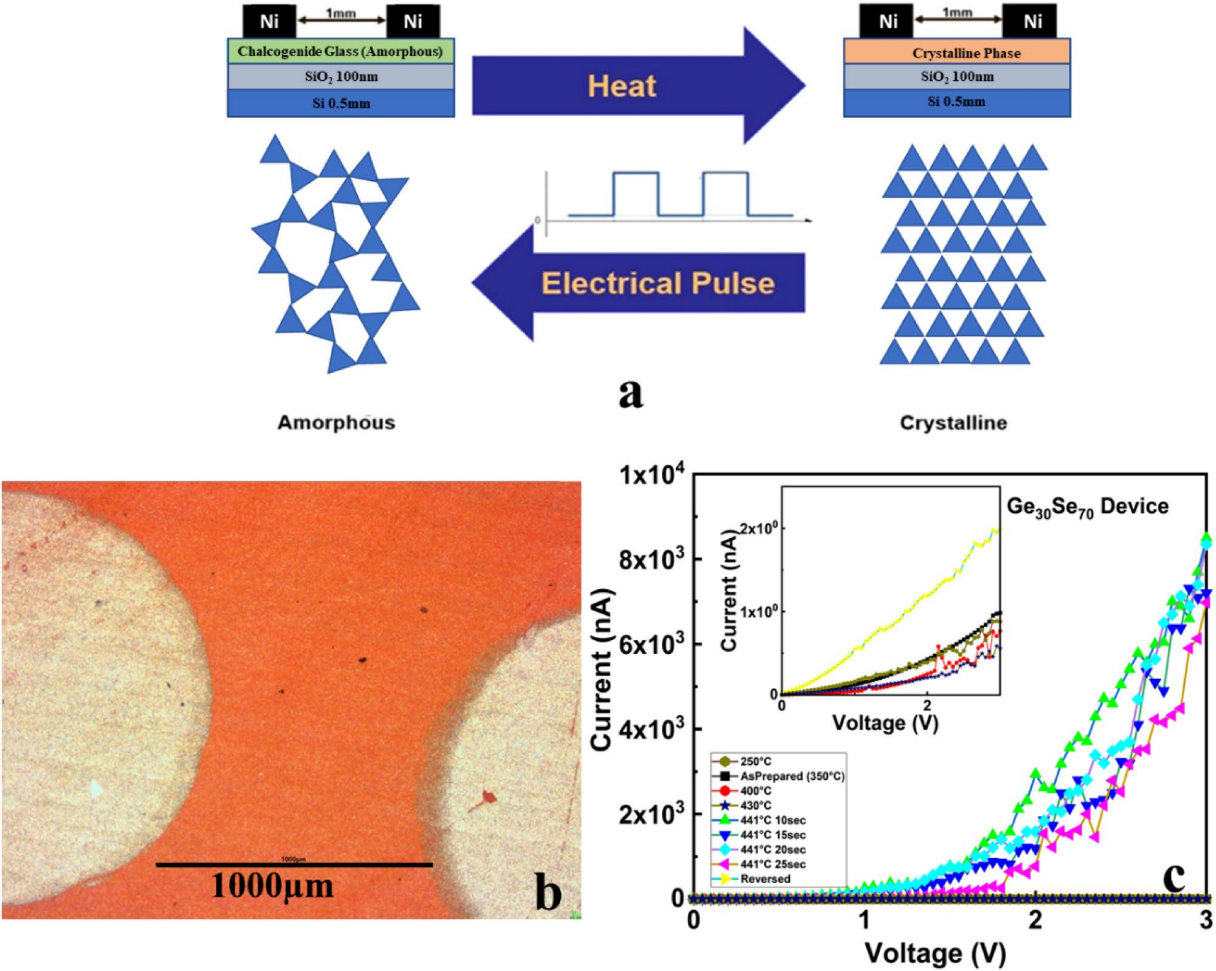
Schematic of a printed device with (**a**) its operational mode, (**b**) optical microscope photo of the device, and (**c**) IV characteristic of the device.

A schematic view of an electronic temperature sensor, device photo, together with its operational mode and IV characteristics, is presented in Fig. 8. The device was made by printing 10 layers (thickness 5 µm) of Ge_30_Se_70_ and the Ni electrodes were placed 1 mm apart by screen printing. The crystallization onset temperature of Ge_30_Se_70_ is 441°C^32^. The device was heated gradually up to the onset of crystallization temperature. At each temperature, the device was kept for 15 s. Before reaching the crystallization temperature, the amorphous material is highly resistive, and there is pA level current flowing through the film. Once the temperature reaches the crystallization onset, a drastic change in I–V characteristic occurs since the material becomes conductive and high current flows through the film. Building an array of devices with different compositions makes real-time monitoring of temperature possible since the crystallization temperature is composition dependent. It is also heating rate dependent which adds one more level of information by application of these sensors. The device was reversed using an electrical pulse, after which the cycle can be repeated, as demonstrated in Fig. 8c. Reversibility was achieved after voltage pulsing by which a Joule heating is produced, which partially melts the film. The final step of the reversing process is a fast cooling of the melted film in contact with the wafer held at room temperature, by which the melt solidifies in amorphous condition. After 10 min of pulsing, the printed device was RESET (10 V square wave, 8 µs period, and 45 ns ON time).

### Optical temperature sensor

**Figure 9:**
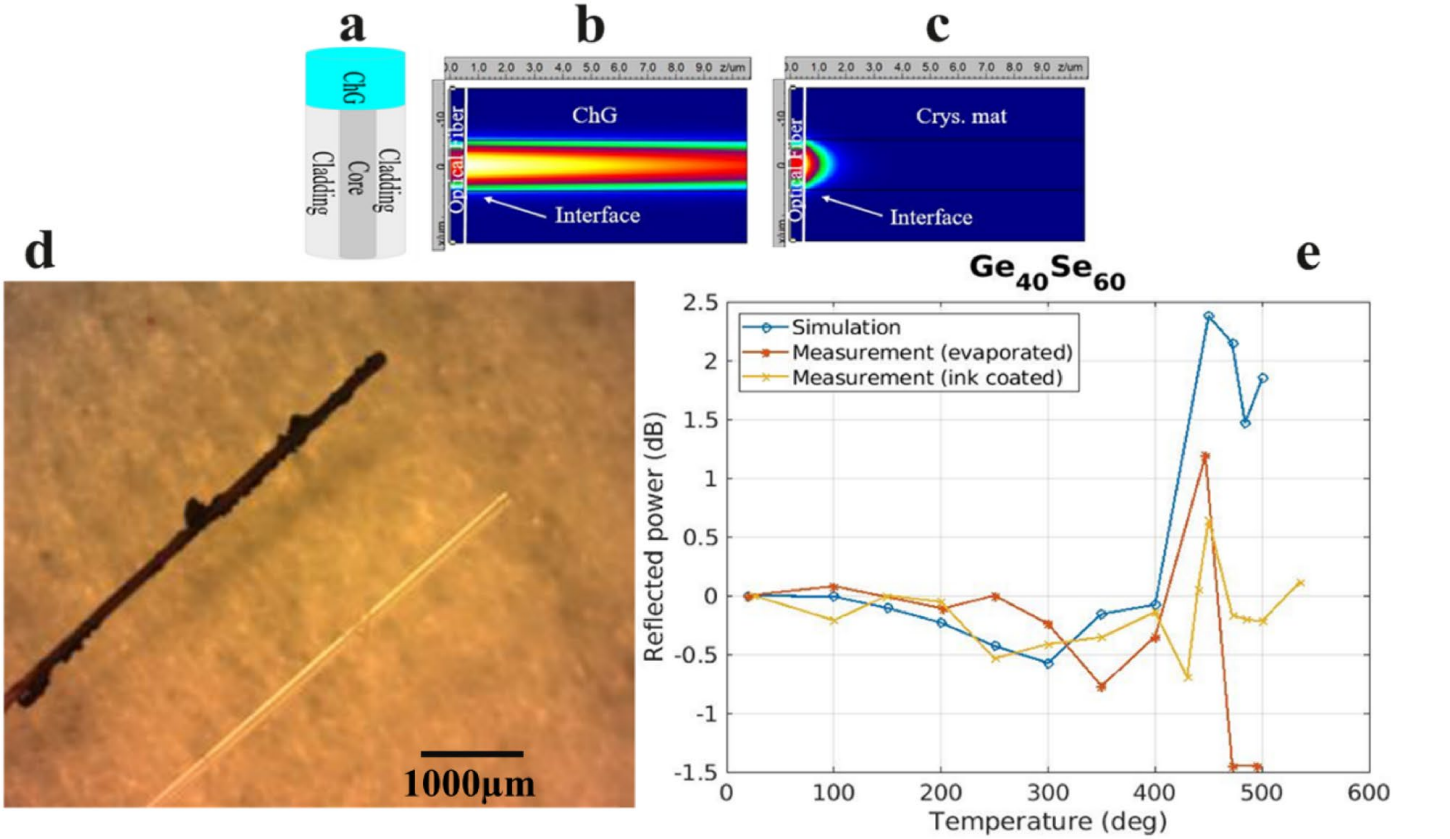
(**a**) Cross-section of ChG-capped optical fiber tip-based temperature sensor; (**b**) power distribution at the fiber-Ge_40_Se_60_ interface in the amorphous phase, (**c**) power distribution at the fiber-Ge_40_Se_60_ interface in a crystalline phase, (**d**) dip coated fiber tip (dark) and blank fiber tip (transparent), (**e**) comparison of sensor performance. (**b**,**c**) Results of simulation done in PhotonDesign (FIMMWAVE, FIMMPROP version 6.6.2) software (http://www.photond.com/index.htm).

A schematic, simulation and performance of an example of a sensor are presented in Fig. 9. The schematic of the optical fiber-based temperature sensor is shown in Fig. 9a. It consists of single-mode optical fiber with capped ChG on the end facet of it. This structure is simulated in PhotonDesign (FIMMWAVE, FIMMPROP version 6.6.2) software, which utilizes a fully vectorial finite-difference mode solver method. For instance, Ge_40_Se_60_ synthesized ink is selected to cover the Rad Hard (Nufern: S1550-HTA) fiber tip with optical properties of n_core_ = 1.44715 and n_cladding_ = 1.45735. The optical properties of synthesized Ge_40_Se_60_ as a function of temperature are measured using Ellipsometer in amorphous and crystalline phases at 1550 nm wavelength. The obtained values in the amorphous phase (n_amor_ = 2.63104 + i0.00575) and crystalline phase (n_crys_ = 3.1099 + i0.211)^32^ are inserted in the model. The study of power distribution in amorphous and crystalline phases shows that in the amorphous phase, when the temperature is below the crystalline temperature of the Ge_40_Se_60_, this composition acts as a dielectric with low absorption and low reflection at the interface of fiber and Ge_40_Se_60,_ as shown in Fig. 9b. By increasing the temperature beyond crystalline temperature (*T* > *T*_c_), the Ge_40_Se_60_ crystallizes and acts like a metal. Then, a higher fraction of the light is reflected into the fiber, and transmitted power inside Ge_40_Se_60_ decays rapidly due to a higher refractive index as well as a higher extinction coefficient. This abrupt change happens at a well-defined temperature which is the crystallization temperature of the particular material. This unique temperature in each of the ChG compositions can provide information regarding the ambient temperature and the heating rate. The sensor is fabricated, and its temperature response as a function of time is measured, as shown in Fig. 9e. The comparison between the simulated normalized reflected power and the measured results matches very well. The sudden change in measured reflected power is observed at the crystallization temperature of Ge_40_Se_60_ (Tc = 472.3 °C). This result confirms that ChGs are highly temperature-dependent and can be used in temperature sensing applications. Reversing of the device to its initial active film amorphous condition is achievable with electrical pulses as illustrated in the previous application example.

## Conclusions

In summary, we describe a nanoparticle ink formulation method of chalcogenide glasses that is compatible with the DMP-2850 printer and can also be applied by dip-coating. The process has been successfully applied to produce 100 nm Ge_x_Se_100−x_ nanoparticles and by modifying additives concentration, DMP-2850 compatible inks were prepared, which have been able to print as low as 100 µm features. The printed thin films were characterized and it was found that Ge_33_Se_67_ nanoparticles crystallized during milling. Ge_30_Se_70_ and Ge_40_Se_60_ printed films have molecular structure and composition, similar to these of the thermally evaporated films. XRD demonstrated, that once crystallized, printed films with x = 30 and x = 40 form orthorhombic structure. The printed thin films have been used to fabricate temperature sensors, proving that the printed films' crystallization temperature is the same as this of thermally evaporated films. Furthermore, dip-coating with the same inks has been successfully applied to coat fiber-tip with a layer of ChG. Dip-coated optical fibers have been heated to crystallize and the performance was compared with simulation and thermally evaporated devices. Thin film devices and optical fiber devices prove that the phase change property and crystallization temperature remain the same in printed films compared to evaporated films. This work enables inkjet printing of ChGs and thus added a versatile family of materials to the ever-growing library of printable materials for microelectronics application.

## Experimental and methods

### Ball milling

The ink formation starts with wet milling of ChG. Before milling, the bulk glasses were crushed into smaller particles by agate mortar. For wet milling, 14 g of ChG, 3 g of ethylcellulose and 50 ml of cyclohexanone were mixed and were introduced into the milling jar. The ball mill is not built for continuous production, so it was programmed to mill for 30 min, then a 30 min pause and repeat. It has the provision to control the temperature during milling. Using that temperature was kept below 50 °C. Milling of ChG was done with 2 mm stainless steel milling balls. Retsch High Energy Ball Mill E_max_ was used for ball milling. It is a state-of-the-art milling system that can mill at 300–2000 rpm and produce nanoparticles < 80 nm. It is programmable, and at a time, two different materials can be milled. The milling rpm was set at 1100 rpm after optimizing by trial and error.

### Ultrasonicating

For ultrasonication, a QSONICA ultrasonicator (500 W) was used. The ultrasonicator's electronic generator transforms AC line power to a 20 kHz signal that drives a piezoelectric transducer. The vibration is then amplified, which transmits down the length of a probe. The tip submerged into the sample expands and contracts. Due to the rapid vibration of the tip, it causes cavitation, the formation and collapse of miniscule bubbles in the liquid. The breakdown of thousands of bubbles releases tremendous energy in the liquid. Objects and surfaces within the liquid are thus "processed". The probe tip diameter dictates the amount of sample that can be effectively processed. Smaller tip diameters (Microtip probes) deliver high-intensity sonication, but the energy is focused within a small, concentrated area. Larger tip diameters can process larger volumes. The ink test tube was put in an ice bath during ultrasonication to prevent heating of the sample. The sonicator parameters were ON/OFF time = 2 s/4 s at 50% power.

### Centrifugation

For centrifugation, a Thermofisher centrifuge system was used at 4500 rpm (maximum) for 1–2 h. It should be stated that when a mixture is placed inside the centrifuge slot, an approximately equally weighted test tube filled with water or any inert material must be placed in precisely the opposite slot of the mixture. It is required to keep balance and stable centrifugation.

### DLS

NanoBrook Omni DLS utilizes light scattering to measure particle size. For the experiment, pure cyclohexanone was poured in a vendor-recommended glass cuvette. Then only a tiny drop of ink was dropped and mixed with the cyclohexanone. DLS needs the light to pass through the cuvette, so it is necessary to use a dust-free cuvette and the amount of ink should be as low as possible.

### Viscosity

A Brookfield AMETEK DV3T Rheometer was used to measure the viscosity of the inks. For final adjustment of the ink viscosity, cyclohexanone and ethylcellulose were added to the milled mixture to prepare a compatible ink. The optimal concentration was found to be 0.15–0.3 g/ml chalcogenide glass and 0.03–0.05 g/ml ethylcellulose in cyclohexanone. The viscosity of the prepared inks was measured and was found to be 10–12 cP, which satisfies the requirements of the DMP-2850 printer.

### Contact angle

The contact angle of the ink on oxidized silicon was measured using an Attention tensiometer. All three ChG compositions showed contact angle 10°–15°, which is suitable for surface wettability. For improvement of the adhesion, the substrate was plasma cleaned.

### Dip-coating

The fiber-tips were dipped in ink under vacuum at room temperature. After 24 h, the tips were carefully taken out of the ink. Then the tips were further cured using a hot chuck in a two-step process: (1) the coated fiber was heated at 50 °C for 2 h to slowly dry the solvent, cyclohexanone, without creating cracks in the film, and (2) the fiber tips were placed in a tube furnace heated at 350 °C for 1 h to decompose the surfactants in ink, ethylcellulose. Once cooled, the fiber tip was dip-coated with spin-on-glass for isolation of the tip from an oxygen-containing ambient. After drying at room temperature for 24 h, the coated fiber was heated at 300 °C for 3 h to cure the spin-on-glass.

### Thin film sintering

After printing, the printed films are wet, and the nanoparticles are mixed with the surfactant. The printed films were dried for 2 days in a vacuum chamber at room temperature for the initial slow solvent evaporation to avoid crack formation. Once dry, the thin films were annealed at 350 °C (the decomposition temperature of ethylcellulose) for 2–3 h. During this time, the particles are sintered and the features are hardened to form solid printed films.

### Raman spectroscopy

Raman analysis was performed in a Horiba LabRAM HR Evolution Raman Spectroscopic System in backscattering mode, using a parallel‐polarized 632.817 nm He:Ne laser, focused to a spot of 6 μm, with a power of 17 mW. Samples were measured at room temperature and under standard atmospheric pressure.

### X-ray diffraction (XRD) spectroscopy

The XRD was done on a Rigaku MiniFlex600 (λ = 1.5406 Å) at 40 kV and 15 mA. At 10 °/min scanning rate, the data were collected at room temperature, in a range of 2θ = 10°–65°.

### Energy dispersive spectroscopy (EDS)

Energy dispersive spectroscopy (EDS), used to confirm the exact composition of the produced films, was conducted using an FEI Teneo Scanning Electron Microscope (SEM) with an Oxford Instruments Energy + EDS system. A line scan of each sample was done at a length of 1500 µm for the collection of an accurate average value and the standard deviation.

### Atomic force microscopy

Surface roughness was characterized via atomic force microscopy (AFM) using a Bruker Dimension FastScan system operating in PeakForce Tapping mode. A ScanAsyst-Air probe with nominal 2 nm radius of curvature and 0.4 N/m spring constant was used to capture a pair of 5 µm × 5 µm topography images at two different locations on each sample surface. The raw images were then processed with a first-order XY plane fit to remove sample tip and tilt using Bruker's NanoScope Analysis software package (Version 2.0) before calculating the root mean square (RMS) surface roughness data presented in Fig. 4.

### Electronic device characterization

The devices were characterized in a semiconductor parametric analyzer (Agilent 4155B). I–V characteristics were measured from 0 to 3 V at a resolution of 30 mV/step. To achieve phase change of initially amorphous active material, the devices were kept for 15 s at each temperature, including the onset of crystallization temperature. Crystallized devices were pulsed with a Pulse Generating Unit (PGU) at different duration for amorphization with square wave amplitude 2 V, period 1 μs, and ON time: 200 ns.

### Optical fiber device characterization

The fabricated sensor devices' performance was characterized by injecting a 1550 nm wavelength light from a tunable laser source into the fiber sensor through a circulator. The light power reflected from the fiber sensor was analyzed using an optical spectrum analyzer (Anritsu MS9740A). The ChG-capped fiber tip itself was placed inside a high temperature-controlled tube furnace (Eurotherm 2116 controller). The furnace temperature was increased from room temperature (~ 25 °C) to 650 °C in 10 °C/min steps. For evaluation of the sensor's real-time response, the temperature inside the furnace as a function of the time was tracked as well.

## Supplementary Information

Supplementary Information 1.
