# Supplementary material for: Introduction of Chalcogenide Glasses to Additive Manufacturing: Nanoparticle Ink Formulation, Inkjet Printing, and Phase Change Devices Fabrication
Source: Sci Rep. 2021 Jul 12;11:14311. doi: 10.1038/s41598-021-93515-y (PMC8275797; doi:10.1038/s41598-021-93515-y)
Supplement: Supplementary file 1 — Supplementary Information 1. [file 41598_2021_93515_MOESM1_ESM.pdf]

Introduction of Chalcogenide Glasses to Additive Manufacturing - Nanoparticle Ink Formulation, Inkjet Printing, and Phase Change Devices Fabrication

A. Ahmed Simon[1], B. Badamchi[1], H. Subbaraman[1], Y. Sakaguchi[2], L. Jones[1], H. Kunold[1], I. van Rooyen[3], *M. Mitkova[1]
[1]Department of Electrical and Computer Engineering, Boise State University, Boise, ID 83725-2075, USA
[2]Comprehensive Research Organization for Science and Society (CROSS)162-1, Shirakata, Tokai, Ibaraki 319-1106, Japan
[3]Idaho National Laboratory, Idaho Falls, ID 83415, USA
*Corresponding Author

Supplementary Information

Coffee-Ring Effect

The ink droplet after drying shows coffee-ring effect. The phenomenon is visible mostly on single droplets.

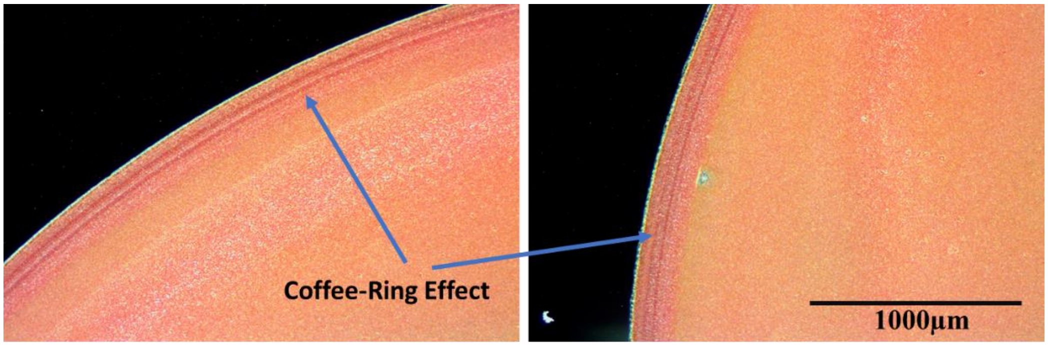

Figure 1 Coffee-Ring effect on ink drop.

Thickness of the as printed films

The thickness was measured using a Dektak stylus profilometer (Fig. 2) . Height of the printed films show 10 layers of printing gives 5-6μm of thickness.

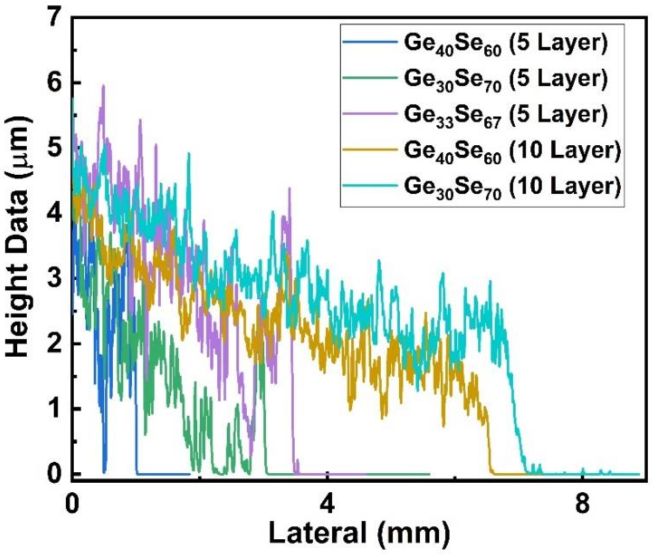

Figure 2 Thickness of the printed films.

Viscosity Measurement

Fig. 3 shows data of viscosity measurement at different speed. The inks have been found to be *shear thinning*.

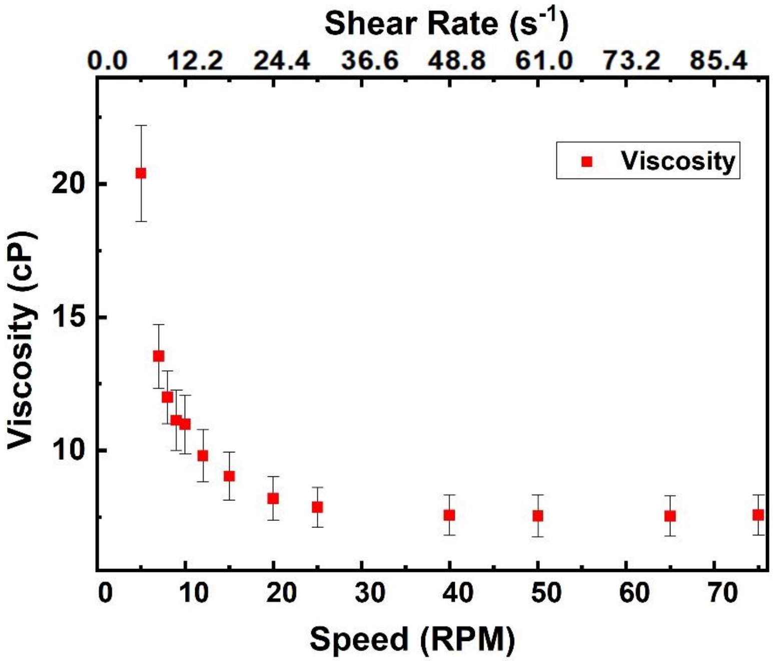

Figure 3 Viscosity of the inks.
